# Industrial Structure Adjustment and Regional Green Development from the Perspective of Mineral Resource Security

**DOI:** 10.3390/ijerph17196978

**Published:** 2020-09-24

**Authors:** You Zheng, Jianzhong Xiao, Jinhua Cheng

**Affiliations:** School of Economics and Management, China University of Geosciences, Wuhan 430074, China; zhengyoyo@cug.edu.cn

**Keywords:** industrial structure adjustment, mineral resource security, green development, Spatial Durbin, Shift-share Method

## Abstract

Mineral resource security is the premise and foundation of the regional green rise strategy. And the adjustment of industrial structure is an effective way to relieve the pressure of the current green economy transformation. Based on the Shift-share Method and the Spatial Durbin model, this paper takes 30 regions in China from 2006 to 2017 as examples to study the impact of industrial structure adjustment on China’s green development from the perspective of mineral resource security. The empirical results show that: China is still in the process of industrial transfer. The dynamic effect of industrial structure promotes green development from the perspective of mineral resource security, while its static effect inhibits green development from the perspective of mineral resource security. The spatial spillover effect of the industrial structure affecting green development from the perspective of mineral resource security is significant. The static structural effect of the tertiary industry promotes the green development of the region, and it has a significant negative impact on neighboring areas, while the secondary industry’s static structural effect has the opposite effect.

## 1. Introduction

At present, China is in a period of slowing economic growth. The original production mode of “big input, big output, high pollution, and high energy consumption” has begun to change to a production mode of “low energy consumption, low pollution, and high efficiency.” The growth mode has also changed from an extensive growth in the quest for speed and scale to intensive growth in pursuit of quality and efficiency. Pleasant ecological environment and sustainability of economic development are inevitable requirements for high-quality development. Green development, which combines economic sustainability with the sustainability of resources and environment, is the critical development path to realize the harmonious unification of economic growth and natural resources and environment [1].

The concept of green development was first put out by Boulding in 1966 for the first time [2]. With global climate change, green development has become the focus topic in academia [3]. Pearce et al. advocated the development of the green economy. They thought the natural ecological environment’s capacity should be fully considered while economy developed [4]. The OECD (2011) regards green development as a solution to the pursuit of economic growth while preventing ecological environment degradation [5]. It is an economic development mode that both covers the limitations of the environmental carrying capacity and strives to achieve sustainable development with environmental protection as one of the main priorities [6]. The research on green development mainly focuses on evaluating the regional green development level and the influencing factors of green development [7,8]. Scholars evaluate the level of regional green development level often by constructing evaluation index systems and data envelopment analysis (DEA) method [9]. Many scholars regarded industrial structure is one of the most important influencing factors on green development through empirical analysis [10,11,12,13]. Due to China’s long-term neglect of environmental costs in industrialization, the transformation of the green economy is facing unprecedented pressure [14]. The adjustment of industrial structure is an effective way to relieve the stress of the current green economy transformation and achieve win-win economic benefits and environmental and resource benefits [15]. The 13th Five-Year Plan for China’s National Economic and Social Development also points out that adjusting industrial structure is an important starting point for achieving green development and an essential element for improving China’s green development efficiency [16].

The industrial structure is usually defined as industrial composition. Therefore, in essence, industrial structure adjustment is defined as the change of industrial structure [17,18]. Significant changes have taken place in China’s industrial structure since 1978. The primary industry and the tertiary industry show a scissor-like symmetrical trend. The tertiary industry has gradually replaced the dominant position of the secondary industry [19]. There are many methods to measure the adjustment of industrial structure, of which a single dimension index, the proportion of secondary industry to Gross Domestic Product (GDP), is the mainstream method widely used [20,21,22]. In recent years, multi-dimensional indicators such as the upgrading and rationalization of the industrial structure have been used to measure industrial structure [23,24,25].

However, industrialization requires large-scale exploit and consumption of natural resources, and its technical route always tends to select abundant reserves materials which are easy to be exploited [26,27,28]. From 1900 to 2005, the global consumption of material increased eightfold, and the dominant resource also changed from the original biological resource to mineral resource [29,30,31]. At present, the world economy has entered in a “new normal era” [32]. The population growth and a new wave of the world’s industrial revolution poses a severe threat to global mineral resource security [33]. Since the 18th Communist Party National Congress of China, the construction of ecological civilization has been incorporated into the overall strategic layout of “five in one” [34]. Mineral resources are an essential material basis for the construction of ecological civilization [35,36]. Future industrial restructuring is bound to affect mineral resource security [16]. Mineral resource security refers to stable, sustainable, economical and adequate access to various minerals under the premise of not destroying the ecological environment [35,37], which is an essential component of regional sustainable development and is also the premise and foundation of regional green development strategy [38].

The above-cite research shows that the industrial structure is the key to improve the level of green development from the perspective of mineral security. Nevertheless, most previous researches have regarded industrial structure as an influential factor of green development; few studies have determined the impact mechanism of industrial structure on green development [38]. At the same time, the mineral resource security should be considered in the process of industrial structure adjustment. However, there are few studies on regional green development from the perspective of mineral resource security. The contribution of this research is mainly in three aspects. First, we describe the effect of structural adjustments on green development using the Shift-share Method, which can evaluate the effect of changes between and within industry sectors on green development [39]. Second, mining is an essential basic industry for national economic development and supporting industry for industrialization [40]. And mineral resource security guarantees the high-quality development of China’s economy. When evaluating the effect of industrial structure on regional green development, mineral resource security is considered. Third, considering the spatial factors, we introduce the spatial Durbin model to study the impact of industrial structure adjustment on green developments.

## 2. Variables and Model Specification

### 2.1. Dependent Variable

The dependent variable is the regional green development level from the perspective of mineral resource security. Green development is essential to obtain the best economic and social benefits with the least material resources consumption and the least environmental cost [41,42]. To carry out the analysis of green development in various regions is a complicated system involving five subsystems, including resources, environment, population, economy, and society. Firstly, combining the basic idea of material flow analysis, this paper selects critical indicators from the above five subsystems as input and output indicators and constructs an evaluation index system of regional green development capability based on the principle of data availability. The selected indicators may affect the evaluation results due to their subjectivity. Therefore, compared with the multi-level evaluation index system method, the DEA method has the main advantage that it can effectively avoid subjective factors in weight setting. Therefore, this paper chooses the DEA model, which is the relative effectiveness evaluation method of a multi-index input/output complex system, to evaluate the green development level from the perspective of mineral resource security in different regions [43].

### 2.2. Core Independent Variable

The core independent variable of this article is the change of industrial structure. In this paper, Shift-share Method is used to measure the transformation of industrial structure. This method is an effective method to reveal the reasons for the change of regional industrial structure, analyze the regional development gap, and determine the future development of leading industries in regular empirical applications [44]. This method is often used by academia to measure changes in industrial structure. It can separate factor productivity according to structural effect and internal growth effect, taking into account changes in employment [45,46]. The analytic expression is:

(1) $$\frac{\Delta P}{P_{0}}=\sum_{i=1}^{n}\frac{S_{i0}\Delta P_{i}}{P_{0}}+\sum_{i=1}^{n}\frac{P_{i0}\Delta S_{i}}{P_{0}}+\sum_{i=1}^{n}\frac{\Delta S_{i0}\Delta P_{i}}{P_{0}},$$

where *i* is the *i* industry, *n* is the number of tertiary industries, *p* is the labor productivity calculated by added value, *s* is the proportion of employed persons, 0 represents the initial value, and Δ represents the change between periods [0, T]. On the left side of the expression is the total labor growth rate; that is, the average labor productivity growth rate during the observation period. The three parts on the right can be regarded as three sources of growth rate. The first part on the right is the intra-group effect or technological progress effect (effects_in), which indicates that the weighted sum of labor productivity growth in the three sectors of the economy. The second part is the static effect or static structural change effect (effects_sta), which indicates the structural change effect caused by the transfer of the employed population in this department to other departments under the condition that the labor productivity level is unchanged. When labor transfers to departments with higher than average productivity, the value of this part is positive; otherwise, it is negative. The third part is a dynamic effect or dynamic structural change effect (effects_dyna), which indicates the combined result of labor transfer and labor productivity change in the employment department. When labor transfers to a sector with higher than average productivity and the industry’s size is expanding, or when the labor force is shifted to an economic sector with lower than average productivity and the size of the sector is shrinking, the value is positive; otherwise, it is negative [47]. The sum of the second part and the third part is the structural effect. The three industries’ structural classification can not only be measured from these three dimensions, the total effect of the three industries’ structural changes can also be obtained by summing up according to the industrial classification.

### 2.3. Control Variables

Through literature collection and collation [48,49,50,51], GDP per capita (GDP_pc_), openness (total import and export trade/GDP, OPEN), urbanization rate (the proportion of the urban population in the region to the total population at the end of the current year, CIT), and technological progress (investment intensity of research and experimental development (R&D), INV) are selected as the basic explanatory variables.

### 2.4. Spatial Doberman Model (SDM)

The extent of the industrial structure affecting green development may be affected by spatial factors [50]. After conducting the LR test (Likelihood-ratio test) between spatial econometric models [52,53,54,55], the SDM model is chosen. The SDM model is used to evaluate the spillover effect of industrial structure adjustment on green development between adjacent provinces:

(2) $$y_{it}=\eta wy_{it}+\alpha x_{it}+\beta wx_{it}+\mu_{it},$$

where *y_it_* represents the explained variable, *x_it_* is the explanatory variable, *w* is the spatial weight coefficient, and *μ_it_* is the random error term; η, α and β are estimated parameters.

### 2.5. DEA Model

DEA model maps the input and output data of decision-making units into coordinate space through mathematical planning to obtain the maximum output boundary or minimum input boundary. It then determines the relative decisions of effective decision-making units and other decision-making units through specific criteria.

## 3. Evaluation of Regional Green Development Competitiveness from the Perspective of Mineral Resource Security

### 3.1. Resource System

Based on energy theory, energy consumption value should be regarded as an essential analysis index of the development degree of mining [56]. In this paper, energy consumption is selected as an input index representing resource endowment, which refers to the sum of raw coal consumption, washed coal consumption, and coke consumption in the process of economic development. It can reflect the different degrees of energy consumption between different regions. The energy consumption can also represent the energy input, which has a significant impact on the industrial chain of mineral resources.

### 3.2. Environmental System

The impact on environmental quality is an important indicator for judging the degree of regional green development when considering mineral resources as energy sources, and the environmental pollutant emissions from material flow analysis can well reflect the environmental output. This paper chooses three indicators to reflect the environmental pollution emissions: industrial wastewater emissions, industrial solid waste emissions, and industrial waste gas emissions to reflect the environmental system [57]. With more emissions, environmental problems get serious. And caused by the production of mineral resources, the region’s green development capacity becomes lower.

### 3.3. Population System

Resources and environmental factors need to be coordinated with human capital factors to develop as a whole, conducive to the production of more social and economic benefits. Therefore, human capital factors are selected in the population system as input indicators of the city’s sustainable development capacity [58,59]. Therefore, this paper selects the number of employees in the secondary industry and the total labor productivity in the population system, which can reflect the size of human input and the efficiency of human capital in the mineral industry, as the input indicators of human capital elements.

### 3.4. Economic System

The advantages of the economic foundation are mainly reflected in the superior market environment, investment environment, ecological environment, and social and economic conditions [60,61]. Therefore, the proportion of investment in extractive industries to investment in fixed assets, and highway mileage are chosen as economic input indicators; the total industrial output value and the average wage of on-the-job workers are chosen as economic output indicators.

### 3.5. Social System

In the transformation process from the advantages of mineral resources to economic advantages, the government has a significant influence and role. It is also one of the decisive indicators to determine whether mineral resources are rationally and optimally allocated. It takes the marketization index as an indicator reflecting the conditions of the social system [62]. Therefore, this paper selects the marketization index as the input index in the social system; the calculation formula for this indicator is:

Marketization Index = (Total Retail Sales of Social Consumer Goods + Sales of Wholesale Goods above Quota)/GDP × 100%(3)

The index system designed in this paper is shown in Table 1, where x1, x5, x6, x7, x10, and x11 are the input indexes of DEA model, and x2, x3, x4, x8, and x9 are the output indexes of the DEA model.

**Figure 1:**
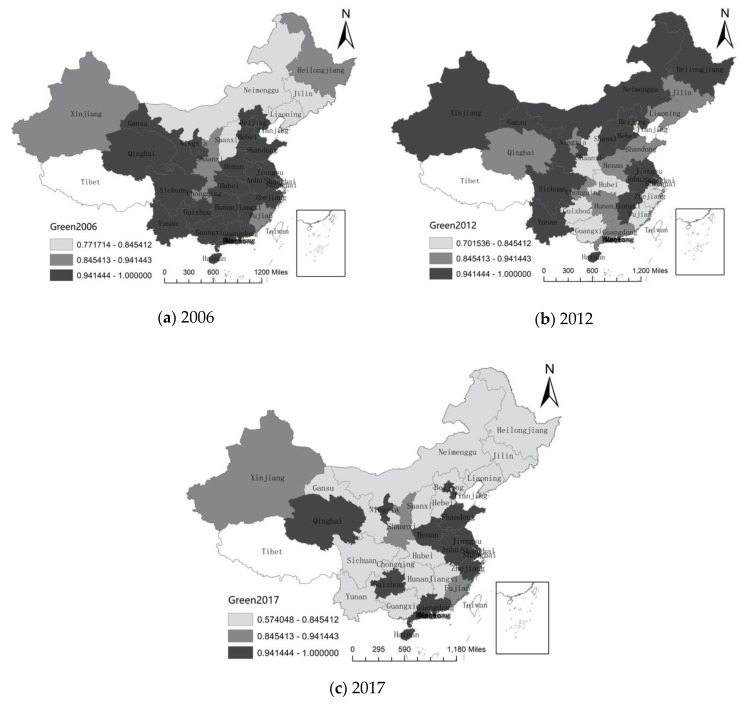
Regional Green Development Level from Perspective of Mineral Security.

The DEA model is used to calculate the regional green development level. According to the nature breaks method, the regions are classified into three categories (Figure 1), darker colors mean higher levels of green development from the perspective of mineral security. Figure 1 can roughly explain the evolution of the level of green development from the perspective of mineral security in China.

Judging from the analysis results, the green development level of mineral resources in various regions shows obvious difference characteristics, which are mainly shown as follows: From 2006–2017, Beijing, Jiangsu Ningxia, Hainan, and Anhui were always in high levels of green development. In Anhui, there were abundant natural mineral resources. In Ningxia and Hainan, the natural environmental conditions are better, and the carrying capacity of resources and environment is relatively strong. In Beijing, while the degree of economic development is high, there are relatively few industries with high demand for mineral resources.

This finding is similar to previous studies [64,65]. The green development level of Heilongjiang, Neimenggu, Shanxi, Jilin, and Liaoning has gone through a process of rising and falling. As the economy further developed, the industrialization process began to accelerate with more pollution and more mineral resource consumption, which resulted in a low green development in 2017. In Gansu, Sichuan, Yunnan, Guangxi, Hunan, Jiangxi, Hubei, Hebei, Xinjiang, Chongqing, the level of green development has experienced varying degrees of decline. It is mostly due to the implementation of the “Western Development” strategy and the “Central China Rise” strategy. Many heavy chemical industries with high energy consumption and high pollution have been transferred to the western regions and central China, resulting in a decrease in the level of green development. From 2006 to 2012, The level of green development in Shanghai also dropped from high to medium level in these years, the increased opening in shanghai to the outside world has attracted more foreign direct investment (FDI), which has led to an increase in resource consumption and has had a negative impact on the environment [66]. However, Qinghai, Guizhou, Shaanxi, Henan, Zhejiang, Shandong, Guangdong, Tianjin, and Fujian developed from a higher level of green development to a low-level green development first, and then developed to a higher level of green development again, just as the results of Ma and Shi [67], which has served as an excellent example for the country’s green development [68]. In general, judging from the regional distribution characteristics, green development level from the perspective of mineral resource security in the central provinces is relatively low, and it is relatively high in China’s eastern coastal regions.

## 4. Spatial Econometrics Results

### 4.1. Benchmark Regression Model and Results

**Figure 2:**
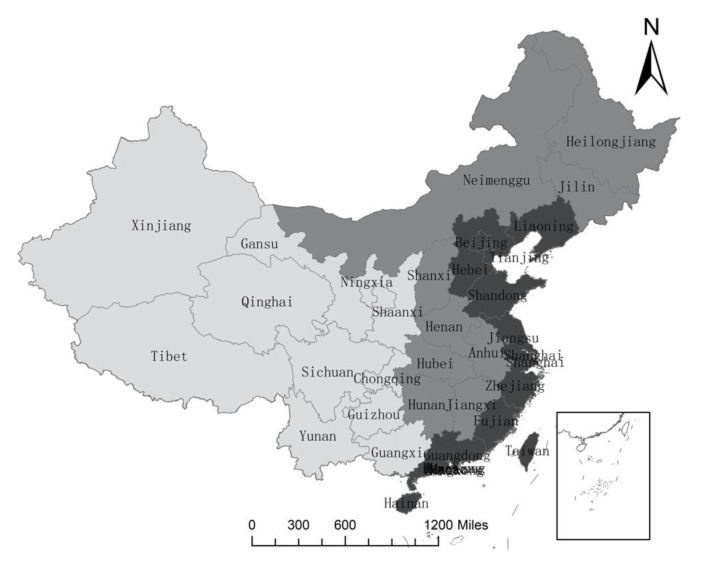
China’s eastern, central and western administrative districts.

Before spatial econometric regression, ordinary least squares (OLS) model, fixed-effect model, and random-effect model are selected for regression to analyze how industrial structure transformation affects green development from the perspective of mineral resources security without considering influential spatial factors. According to the industrial structure of the three industries in national, eastern, central, and western regions (Figure 2, from left to right, the three-color blocks represent the western, central and eastern regions), the benchmark regression model is as follows:

(4) $$y_{it}=\beta_{1}effects\text{\_}in_{it}+\beta_{2}effects\text{\_}sta_{it}+\beta_{3}effects\text{\_}dyna_{it}+\beta_{4}openness_{it}+\beta_{5}cit_{it}+\beta_{6}GDPpc_{it}+\beta_{7}inn_{it},$$

Table 2 shows the estimation results of the benchmark model, and the Hausman test is carried out. The results show that the fixed-effect model should be selected. It also shows the regression results of the fixed-effect model for the whole region in Table 2, respectively. It can be seen that the regression results are quite different in different regions. Model 1 is the fixed-effect benchmark model taking the overall change of industrial structure as the explanatory variable, and Model 2, Model 3, Model 4 are fixed-effect benchmark models taking the structural changes of the primary industry, the secondary industry and the tertiary industry as the explanatory variables, respectively.

From the national perspective, only the dynamic effect of the industrial structure plays a significant role in promoting green development. The employment changes brought about by the upgrading of industrial structure will affect green development. The static structural effect plays a negative role in green development. The dynamic structural effect of the secondary industrial structure will inhibit green development, while the dynamic effect of the tertiary industrial structure will promote green development. In terms of controlling variables, economic development plays a role in promoting green development. Economic development brings about scale effect and technological advantages, which is conducive to improving the efficiency of the green economy [69]. However, urbanization brings the population agglomeration and aggravating pollution, which has a negative effect on green development. The technological progress effect of the secondary industry can promote green development. Simultaneously, the increase in employment and the expansion of departments will reduce the degree of green development in the region. When the scale of the primary industry shrinks and the tertiary industry scale increases, the degree of green development increases.

According to the classification of eastern, central, and western regions (Table 3, Table 4 and Table 5), the regression results show more regional characteristics. As for the intra-group effect of industrial structure, the regression results of the east, middle and west regions are consistent with the nationwide regression results, i.e., technological progress in the industry has a significant promotion effect on green development. The increase in employment in the secondary industry will inhibit green development in the western region and affect local mineral resource security. However, the increase in employment in the tertiary industry will inhibit the green development in the east, which shows that when the tertiary industry develops to a certain extent, the influx of excessive employment in the tertiary industry will affect the local environment.

### 4.2. Regression Results of Spatial Regression Model

The collinearity test of panel data shows that there is no multicollinearity. Then the Moran test is carried out, and the spatial correlation of green development level from the perspective of mineral resource security is significant. In order to investigate whether the explained variables in neighboring provinces are spatially dependent, and whether the industrial structure changes will have spillover effects on green development of adjacent areas, this paper, based on various tests, selects the Spatial Doberman Model (SDM) to continue to discuss this problem.

Through the Hausman test, the spatial Durbin model with time and space double fixed is selected (in Model 5, the explanatory variable is the overall change of industrial structure. In Model 6, Model 7, and Model 8, the explanatory variables are the structural changes of the primary industry, the secondary industry and the tertiary industry, respectively.). As can be seen from Table 6, the dynamic structural effect of the industrial structure plays a promoting role in green development. The employed population is transferred from the primary industry to the secondary industry and then to the tertiary industry. With technology developed rapidly, the industrial-scale increases, the degree of green development increases, the safety of mineral resources increases, and the primary industry’s significant dynamic structural effect hinders green development. Increasing the static structural effect of the secondary industry also hinders green development. According to Petty-Clark Law, when the employed population shifts from the primary industry to the secondary industry, the consumption of mineral resources will increase, which will lead to a reduction in the level of green development. When the intra-group effect of the secondary industry and the tertiary industry increases, technology develops, the degree of green development increases, and the degree of mineral resource security increases. In terms of controlling variables, economic development, and technological innovation are crucial elements to promote green development and safeguard mineral resources.

According to the regression results in Table 7, Table 8 and Table 9, the structural effect of the three industries in the eastern region has a more significant impact on the green development from the perspective of the security of mineral resources. 

For the eastern regions, the intra-group effect and scale effect of the secondary industrial structure may improve the local green development level, while the intra-group effect of the tertiary industrial structure has a reduction effect on the local green development level. 

For the central and western regions, the intra-group effect and scale effect of the secondary industrial structure reduce the local green development level, while the intra-group effect of the tertiary industrial structure promotes the local green development. As the eastern region has entered the late stage of industrialization, in these regions, balanced development among the three industries should be paid more attention, and the development of the tertiary industry should not be blindly supported. However, in the central and western regions, the industrial structure should be further adjusted to improve the local green development level.

As for the control variables, opening to the outside world promotes the green development in eastern and central regions. However, it will reduce the level of green development in the western region and affect local mineral resource security. This is most closely related to the different economic development stages in the eastern, central, and western regions. In eastern regions, the economic development degree is the highest, so the opening degree of the region is more used for the development of local technology, which can effectively reduce environmental pollution and ensure the safety of mineral resources. However, in the western region, the level of economic development and openness is the lowest. It is still in the middle stage of industrialization. Increasing the degree of openness in this region will lead to the introduction of more polluting enterprises, more consumption of local mineral resources, causing local environmental pollution and lowering the level of local green development. The conclusion of this part is similar to that of Ru [70].

Spillover effect can be decomposed by partial differential method into direct effect and indirect effect [71]. The spatial spillover effect of industrial structure transformation on green development is significant. Judging from the three branch effects of industrial structure, the direct effect is the influence on the dependent variable in the region, including the “feedback effect”, that is, the influence of neighboring pairs will be fed back to the region. And the indirect effect is the effect on the neighboring regions.

As can be seen from Table 10 and Table 11, the intra-group effect results of different industries in each model are negative at the national level, consistent with the above observation and analysis. The static structural effect of the tertiary industry promotes the green development of the region. It has a significant negative effect on the neighboring regions, while the secondary industry’s static structural effect is the opposite. This result shows that China is currently undergoing employment conversion in the secondary and tertiary industries. The degree of development of the tertiary industry affects the local green development level, thus affecting the safety of local mineral resources, but the development of the tertiary industry is also a significant constraint on the green development of the region at this stage. From a regional perspective, the industrial structure direct effects of the primary and the secondary in the eastern region are very significant. The static and dynamic effects of the two industries are in opposite directions. It shows that the upgrading of industrial structure in the eastern region has a significant impact on local green development. In the eastern regions, when labor transfers to the secondary and tertiary industries from the primary industry, the secondary industry scale expands as well as technology developing, which can effectively improve the efficiency of mineral employ. However, the impact mechanism of industrial structure upgrading on green development in the central and western regions is more complicated due to the slow industrial development and the continuous transportation of labor to the eastern regions. At the national level, the intra-group effect results of different industries in each model are negative. This result shows that China is currently in the process of employment conversion in the secondary and tertiary industries, too.

## 5. Conclusions

In this study, the Shift-share Method is used to measure the adjustment of industrial structure. The degree of green development is measured from the perspective of mineral resource security. Based on the Spatial Durbin model, this paper takes 30 provinces in China from 2006 to 2017 as examples to study the impact of industrial structure adjustment on China’s green development and its spatial spillover effect from the perspective of mineral resource security. The research conclusion is as follows.

First, from 2006 to 2017, the number of regions with a high level of green development from the perspective of mineral resource security decreased in overall of China, and then increased in east China, and most areas of central China experiences the decline of green development level from the perspective of mineral resource security. China is still in the process of industrial transfer.

Second, the dynamic effect of industrial structure promotes green development from the perspective of mineral resource security, while its static effect inhibits green development from the perspective of mineral resource security. The industrial structure cannot be adjusted solely through the allocation of resources. While promoting the transition of production factors from surplus departments to insufficient departments, independent technology innovation should be emphasized.

Third, the intra-group effect and scale effect of the secondary industrial structure in the eastern region enhance the green development level of local mineral resource security, while the intra-group effect of the tertiary industrial structure reduces the local green development level. For the central and western regions, the intra-group effect and scale effect of the secondary industrial structure reduce the green development degree of the local mineral resource security. The intra-group effect of the tertiary industrial structure enhances local green development. Local governments should formulate differentiated strategies and corresponding policies and measurements for industrial structure adjustment according to the current industrial development situation and local comparative advantages.

Fourth, the spatial spillover effect of green development under mineral resource security is significant. The static structural effect of the tertiary industry promotes the green development of the region. It has a significant negative impact on neighboring regions, while the static structural effect of the secondary industry has the opposite effect. At present, China is in the process of employment conversion in the secondary and tertiary industries. When formulating industrial structure adjustment policies, based on the heterogeneity of the local and neighboring regions, the development of green industries should be incorporated into the local and adjacent regions’ industrial development outlines and government officials’ performance appraisal systems. On this basis, through increasing publicity or granting subsidies by the government, green technological innovation and its industrial research should be encouraged; the conversion rate of green technological achievements should be promoted, and consumers should be encouraged to use more green products, to realize the development of green emerging industries without affecting the green development of surrounding areas. Furthermore, the upgrading of traditional local industries is going to accelerate to guarantee mineral resource security.

## Figures and Tables

**Table 1:** Evaluation of Regional Green Development Ability.

Evaluation Index	Variables
Mineral Resources Endowment	Energy Consumption (x1)
Environmental Protection	Industrial Wastewater Discharge (x2)
	Industrial Solid Waste Emissions (x3)
	Industrial Waste Gas Emissions (x4)
Human Capital	Number of Employees in the Secondary Industry (x5)
	Total Labor Productivity (x6)
Economic Base	The proportion of Investment in Mining Industry to Investment in Fixed Assets (x7)
	Total Industrial Output Value (x8)
	Average Wage of On-the-job Workers (x9)
Policy Factors	Highway Mileage (x10)
	Marketization Index (x11)

Note: The three tertiary indicators under environmental protection are all negative input indicators in DEA input indicators, so converting the pollutants in the original data into common expected output is necessary. This paper adopts the commonly used linear data transformation method [63,64], i.e., f(b) = v − b, where b is the original index data. v is a vector large enough to ensure that all converted expected outputs are positive, and then the converted data is added to the DEA model as expected outputs.

**Table 2:** Regression Results of National Benchmark Model.

Variables	Model 1	Model 2	Model 3	Model 4
effects_in	0.0027 (0.0027)	−0.0019 (0.0009)	0.0899 *** (0.0311)	−0.1265 (0.0165)
effects_sta	−0.4676 (0.4888)	0.3260 (0.1666)	−0.4158 * (0.0774)	−0.8153 (0.2039)
effects_dyna	0.3573 *** (0.0930)	−0.2157 *** (0.0573)	0.2284 (0.0155)	0.2549 *** (0.0417)
INN	0.0167 (0.0161)	0.0232 (0.0159)	−0.0030 (0.0155)	0.0528 *** (0.0150)
CIT	−1.2461 *** (0.1205)	−1.2433 *** (0.1346)	−1.3503 *** (0.1278)	−0.5917 *** (0.1262)
GDPpc	0.0101 * (0.0041)	0.0176 *** (0.0038)	0.0093 * (0.0050)	0.0284 *** (0.0042)
OPEN	0.2333 (0.1556)	0.2294 (0.1568)	0.0567 (0.1559)	0.0935 (0.1442)

Note: in the table ***, and * mean significant at the levels of 1%, and 10%, respectively. The brackets are standard deviation.

**Table 3:** Regression Results of Benchmark Model in Eastern Regions.

Variables	Model 1	Model 2	Model 3	Model 4
effects_in	0.002 (0.003)	0.004 ** (0.002)	0.078 (0.050)	0.124 *** (0.023)
effects_sta	0.539 (0.687)	0.516 (0.345)	0.672 (0.437)	0.777 ** (0.299)
effects_dyna	0.186 (0.149)	−0.543 (0.177)	−0.184 (0.182)	0.243 *** (0.061)
INN	−0.012 (0.025)	−0.011 (0.022)	−0.008 (0.024)	0.029 (0.023)
CIT	−1.076 *** (0.191)	−1.441 *** (0.243)	−1.298 *** (0.233)	−0.393 * (0.201)
GDPpc	0.013 * (0.007)	0.020 *** (0.005)	0.162 * (0.008)	0.029 *** (0.006)
OPEN	0.178 (0.172)	0.127 (0.167)	0.124 (0.169)	0.121 (0.152)

Note: in the table ***, **, and * mean significant at the levels of 1%, 5%, and 10%, respectively. The brackets are standard deviation.

**Table 4:** Regression Results of National Benchmark Model in Central Regions.

Variables	Model 1	Model 2	Model 3	Model 4
effects_in	0.206 ** (0.096)	0.216 *** (0.035)	0.031 (0.062)	0.125 *** (0.039)
effects_sta	−2.119 (1.327)	0.596 (0.371)	−0.143 (0.689)	−2.372 (0.454)
effects_dyna	0.793 *** (0.196)	−0.513 (0.194)	0.236 (0.191)	0.574 *** (0.105)
INN	0.153 *** (0.328)	0.138 *** (0.029)	0.108 *** (0.040)	0.152 *** (0.031)
CIT	−1.277 *** (0.315)	−1.136 *** (0.237)	−1.177 *** (0.276)	−1.390 *** (0.281)
GDPpc	0.029 (0.022)	0.030 *** (0.011)	−0.016 (0.014)	0.035 *** (0.013)
OPEN	5.513 *** (0.898)	4.471 *** (0.880)	4.090 *** (1.101)	5.627 *** (0.919)

Note: in the table ***, and ** mean significant at the levels of 1%, and 5%, respectively. The brackets are standard deviation.

**Table 5:** Regression Results of National Benchmark Model in Western Regions.

Variables	Model 1	Model 2	Model 3	Model 4
effects_in	0.143 * (0.084)	0.049 * (0.028)	−0.408 (0.070)	0.096 *** (0.025)
effects_sta	−2.015 (0.705)	−0.486 (0.197)	−0.886 *** (0.299)	−0.756 *** (0.273)
effects_dyna	0.891 *** (0.151)	−0.431 *** (0.088)	0.440 *** (0.093)	0.325 *** (0.057)
INN	0.193 *** (0.036)	0.111 *** (0.038)	0.109 *** (0.036)	−0.117 (0.034)
CIT	−2.099 *** (0.358)	−2.436 *** (0.299)	−2.430 *** (0.220)	−1.938 *** (0.262)
GDPpc	0.091 *** (0.013)	0.086 *** (0.008)	0.083 *** (0.014)	0.084 *** (0.009)
OPEN	−0.600 (0.147)	−0.504 (0.417)	−1.226 ** (0.487)	−0.208 (0.428)

Note: in the table ***, **, and * mean significant at the levels of 1%, 5%, and 10%, respectively. The brackets are standard deviation.

**Table 6:** Results of National Spatial Regression Model.

Variables	Model 5	Model 6	Model 7	Model 8
effects_in	0.003 (0.002)	−0.002 (0.001)	0.087 *** (0.027)	0.034 * (0.018)
effects_sta	−0.595 (0.486)	0.020 (0.132)	−0.785 *** (0.194)	0.249 (0.209)
effects_dyna	0.524 *** (0.102)	−0.192 *** (0.046)	0.292 (0.063)	0.075 (0.047)
INN	0.040 *** (0.013)	0.035 *** (0.013)	0.029 ** (0.012)	0.040 *** (0.013)
CIT	−0.005 (0.137)	−0.134 (0.140)	−0.240 (0.155)	0.075 (0.142)
GDPpc	0.037 *** (0.004)	0.047 *** (0.004)	0.036 ** (0.005)	0.042 *** (0.004)
open	−0.063 (0.127)	−0.095 (0.126)	−0.025 (0.123)	−0.056 (0.125)

Note: in the table ***, **, and * mean significant at the levels of 1%, 5%, and 10%, respectively. The brackets are standard deviation.

**Table 7:** Results of Spatial Regression Model in Eastern Regions.

Variables	Model 5	Model 6	Model 7	Model 8
effects_in	0.008 *** (0.003)	−0.008 * (0.002)	0.327 *** (0.038)	−0.055 * (0.030)
effects_sta	−2.869 (0.966)	0.196 (0.366)	0.965 *** (0.321)	−0.279 (0.331)
effects_dyna	1.217 *** (0.194)	−0.856 (0.199)	−0.455 (0.142)	0.422 *** (0.078)
INN	0.086 *** (0.019)	0.049 *** (0.017)	0.075 *** (0.016)	0.102 *** (0.019)
CIT	0.260 (0.236)	−1.259 *** (0.235)	−0.785 *** (0.235)	0.215 (0.199)
GDPpc	0.020 *** (0.005)	0.041 *** (0.005)	0.077 (0.005)	0.023 *** (0.005)
OPEN	0.205 (0.134)	0.574 (0.120)	−0.039 (0.110)	0.252 * (0.132)

Note: in the table ***, and * mean significant at the levels of 1%, and 10%, respectively. The brackets are standard deviation.

**Table 8:** Results of Spatial Regression Model in Central Regions.

Variables	Model 5	Model 6	Model 7	Model 8
effects_in	−0.185 (0.103)	−0.040 (0.070)	−0.123 *** (0.070)	0.050 (0.052)
effects_sta	−2.782 (1.513)	−0.896 (0.581)	−1.116 (0.787)	−0.773 (0.716)
effects_dyna	1.021 *** (0.333)	0.247 (0.383)	0.546 *** (0.204)	0.504 ** (0.247)
INN	0.084 * (0.046)	0.151 *** (0.046)	0.063 (0.043)	0.114 *** (0.042)
CIT	0.565 (0.521)	−0.313 (0.588)	1.416 ** (0.600)	0.508 (0.489)
GDPpc	0.122 *** (0.004)	0.047 *** (0.015)	0.091 *** (0.029)	0.039 *** (0.015)
OPEN	1.964 * (1.085)	3.453 *** (1.036)	0.950 (1.137)	3.370 *** (0.976)

Note: in the table ***, **, and * mean significant at the levels of 1%, 5%, and 10%, respectively. The brackets are standard deviation.

**Table 9:** Results of Spatial Regression Model in Western Regions.

Variables	Model 5	Model 6	Model 7	Model 8
effects_in	−0.021 (0.074)	0.0348 (0.028)	−0.112 ** (0.054)	0.057 ** (0.028)
effects_sta	−0.575 (0.687)	−0.273 (0.214)	−0.499 ** (0.247)	−0.102 (0.268)
effects_dyna	0.425 ** (0.169)	0.011 (0.112)	0.257 *** (0.727)	0.202 *** (0.064)
INN	−0.164 * (0.032)	−0.122 *** (0.032)	−0.108 *** (0.030)	−0.196 *** (0.034)
CIT	−0.013 (0.426)	−0.171 (0.412)	−0.294 (0.430)	0.303 (0.337)
GDPpc	0.095 *** (0.012)	0.106 *** (0.083)	0.121 *** (0.011)	0.109 *** (0.007)
OPEN	−1.767 *** (0.381)	−0.719 *** (0.647)	−1.177 *** (0.438)	−1.850 *** (0.392)

Note: in the table ***, **, and * mean significant at the levels of 1%, 5%, and 10%, respectively. The brackets are standard deviation.

**Table 10:** Direct effect.

National Regions Variables	Model 5	Model 6	Model 7	Model 8
effects_in	0.008 *** (0.003)	−0.002 *** (0.001)	0.787 *** (0.027)	−0.032 * (0.018)
effects_sta	−2.701 *** (0.931)	0.001 (0.129)	−0.724 *** (0.189)	0.235 (0.204)
effects_dyna	1.161 *** (0.100)	−0.185 *** (0.045)	0.280 *** (0.062)	0.074 (0.046)
**Eastern Regions**				
effects_in	0.008 *** (0.003)	−0.008 *** (0.002)	0.279 *** (0.037)	−0.046 (0.031)
effects_sta	−2.701 *** (0.932)	0.233 (0.372)	0.969 (0.319)	−0.258 (0.311)
effects_dyna	1.161 *** (0.190)	−0.836 *** (0.220)	−0.493 *** (0.149)	0.390 *** (0.073)
**Central Regions**				
effects_in	−0.090 (0.126)	−0.038 (0.070)	−0.091 (0.065)	0.048 (0.052)
effects_sta	−2.523 * (1.510)	−0.891 (0.567)	−0.865 (0.729)	−0.804 (0.666)
effects_dyna	0.954 *** (0.318)	0.276 (0.373)	0.483 ** (0.190)	0.504 ** (0.230)
**Western Regions**				
effects_in	0.038 (0.698)	0.021 (0.026)	−0.063 (0.052)	0.066 ** (0.027)
effects_sta	−0.491 (0.623)	−0.238 (0.192)	−0.382 * (0.229)	−0.109 (0.217)
effects_dyna	0.431 *** (0.150)	−0.042 (0.101)	0.234 *** (0.072)	0.194 *** (0.053)

Note: in the table ***, **, and * mean significant at the levels of 1%, 5%, and 10%, respectively. The brackets are standard deviation.

**Table 11:** Indirect effect.

National Regions Variables	Model 5	Model 6	Model 7	Model 8
effects_in	0.004 (0.005)	−0.002 (0.002)	0.244 *** (0.072)	0.096* (0.049)
effects_sta	−2.824 * (1.228)	1.325 (0.322)	−1.741 *** (0.429)	−0.424 (0.565)
effects_dyna	0.233 (0.251)	−0.261 ** (0.107)	0.460 *** (0.130)	−0.255 ** (0.126)
**Eastern Regions**				
effects_in	0.006 (0.005)	−0.002 (0.003)	0.492 *** (0.083)	−0.087 * (0.047)
effects_sta	−2.937 ** (1.263)	−0.592 (0.4666)	−0.127 (0.391)	−0.393 (0.429)
effects_dyna	0.999 *** (0.366)	−0.091 (0.313)	0.478 *** (0.178)	0.430 *** (0.135)
**Central Regions**				
effects_in	−0.521 * (0.309)	0.008 * (0.119)	−0.323 ** (0.126)	0.057 (0.072)
effects_sta	−1.990 (2.377)	−0.566 (0.927)	−2.941 ** (1.275)	0.012 (1.097)
effects_dyna	0.655 (0.514)	0.049 (0.640)	0.890 ** (0.350)	0.778 ** (0.395)
**Western Regions**				
effects_in	−0.188 (0.192)	0.156 ** (0.064)	−0.505 *** (0.171)	−0.062 (0.059)
effects_sta	−1.484 (1.950)	−0.469 (0.478)	−1.315 * (0.716)	−0.085 (0.795)
effects_dyna	0.075 (0.470)	0.626 ** (0.250)	0.312 (0.218)	0.138 (0.170)

Note: in the table ***, **, and * mean significant at the levels of 1%, 5%, and 10%, respectively. The brackets are standard deviation.
